# Facile Growth of High-Yield Gold Nanobipyramids Induced by Chloroplatinic Acid for High Refractive Index Sensing Properties

**DOI:** 10.1038/srep36706

**Published:** 2016-11-14

**Authors:** Caihong Fang, Guili Zhao, Yanling Xiao, Jun Zhao, Zijun Zhang, Baoyou Geng

**Affiliations:** 1 College of Chemistry and Materials Science, The Key Laboratory of Functional Molecular Solids, Ministry of Education, Anhui Laboratory of Molecular-Based Materials, Anhui Normal University, Wuhu in Anhui Province 241000 China

## Abstract

Au nanobipyramids (NBPs) have attracted great attention because of their unique localized surface plasmon resonance properties. However, the current growth methods always have low yield or suffer tedious process. Developing new ways to direct synthesis of high-yield Au NBPs using common agents is therefore desirable. Here, we employed chloroplatinic acid as the key shape-directing agent for the first time to grow Au NBPs using a modified seed-mediated method at room temperature. H_2_PtCl_6_ was added both during the seed preparation and in growth solution. Metallic Pt, reduced from chloroplatinic acid, will deposit on the surface of the seed nanoparticles and the Au nanocrystals and thus plays a critical role for the formation of Au NBPs. Additionally, the reductant, precursor, and surfactant are all cheap and commonly used. Furthermore, the Au NBPs offer narrow size distribution, two sharp tips, and a shared basis. Au NBPs therefore show much higher refractive index sensitivities than that of the Au nanorods. The refractive index sensitivities and lager figure of merit values of Au NBPs exhibit an increase of 63% and 321% respectively compared to the corresponding values of Au nanorod sample.

Gold nanocrystals (NCs) have been intensively investigated during the past several decades due to their great potential applications in biological science, catalysis, electronics, and photonics^1^,^2^. Most of those applications are on the basis of their intriguing localized surface plasmon resonance (LSPR) features. For Au NCs, their LSPR properties can be synthetically tailored by tuning their shapes and sizes. For example, Au NCs with spherical shape possess an LSPR ranging from 500–800 nm as their sizes were changed from 20 to 220 nm^3^, limiting their application in near-infrared region. In this aspect, anisotropic Au NCs offer more significant advantages. Au nanorods, as an important example, exhibit two plasmon modes including a transverse mode and a longitudinal mode. The intriguing longitudinal LSPR can even be tailored from 580 to 1200 nm covering the range from visible to NIR region by tuning their aspect ratios^4^,^5^. Although the excellent LSPR properties the Au nanorods have, they still have several shortcomings. The round ends of Au nanorods show a relative small localized electric field enhancement. Besides, Au nanorods have a larger size distributions, causing the broadened longitudinal LSPR with the full width at half maximum (FWHM) values in the range of ~150 to 200 nm. Au nanobipyramids (NBPs), composed by two pyramids connected at their bases, are another anisotropic nanostructures that simultaneously possess the sharp tips and synthetically tunable longitudinal LSPRs. Sharp tips of Au NBPs endow them with localized electric field enhancements several times higher than Au nanorods^6^. Another intriguing properties of Au NBP is their high monodisperdity, giving narrower FWHM about 135 eV^7^. Furthermore, the longitudinal LSPR of Au NBPs can be synthetically with a even low-energy wavelength of 1330 nm^8^. The above features make Au NBPs show much more sensitive to the surrounding environment. They therefore perform higher refractive index sensitivities (RIS) and lager figure of merit (FOM) values compared to that of Au nanorods^9^. Great efforts have been subsequently made to prepare Au NBPs. Seed-mediated method is a typical way. Guyot-Sionnest *etc* first synthesized Au NBPs using a typical seed-mediated way, in which small sized Au nanoparticles were used as seeds^10^. However, the relative low yield limits their applications. Wang’s group improved such classic seed-mediated method, in which Au nanoparticles smaller than 5 nm with twin-structure as seeds, Ag^+^ as the shape-directing agents, ascorbic acid (AA) and cetyltriethylammonium bromide are as the reduced and surfactant agents, respectively^11^. However, the synthesis of surfactants is very time-consumed and will inevitably make the synthetic process much tedious. Therefore, it is apparent that there is still an active motivation for developing new ways to direct synthesis of Au NBPs. Until recently, some new methodologies were developed based on seed-mediated method^12^,^13^. For example, Huang *et al*. produced Au NBPs using an overgrowth process by employing large sized Au decahedra as the seeds^14^,^15^. However, this synthetic process involved a high temperature and complex organic agents, making it still difficult to prepare. In most of the synthetic routine, Ag^+^ were commonly employed to direct the anisotropic growth based on underpotential deposition mechanism^16^. However, the deposition behavior of metal ions dependent on their physicochemical properties, such as atomic radii and bond dissociation energies^17^. Theoretically, the morphologies will change if different foreign metal ions are introduced during synthetic process due to their different deposition behaviors. However, there are still rarely reported on this shape-control manner, except silver ions, during the growth of Au NCs especially for Au NBPs^18^.

In this context, we firstly introduced H_2_PtCl_6_ as a key shape-directing agent to grow Au NBPs through a modified seed-mediated method. Cetyltrimethyammonium bromide (CTAB), a common used and cheap surfactant, serves as surfactant during the whole synthetic process. AA was employed as reductant. Au NBPs with yield higher than ~84.6% were prepared. Moreover, we also investigated the synthetic parameters during growth process, involving the effect of the ratio of H_2_PtCl_6_ to HAuCl_4_ in seeds and growth solution, NaBH_4_ during seed preparation, the role of AgNO_3_, and the effect of surfactant in growth solution. Our Au NBPs have a relative narrow FWHM of 59 nm. Furthermore, our Au NBPs show much higher RIS than that of the corresponding Au nanorods.

## Results and Discussion

**Figure 1:**
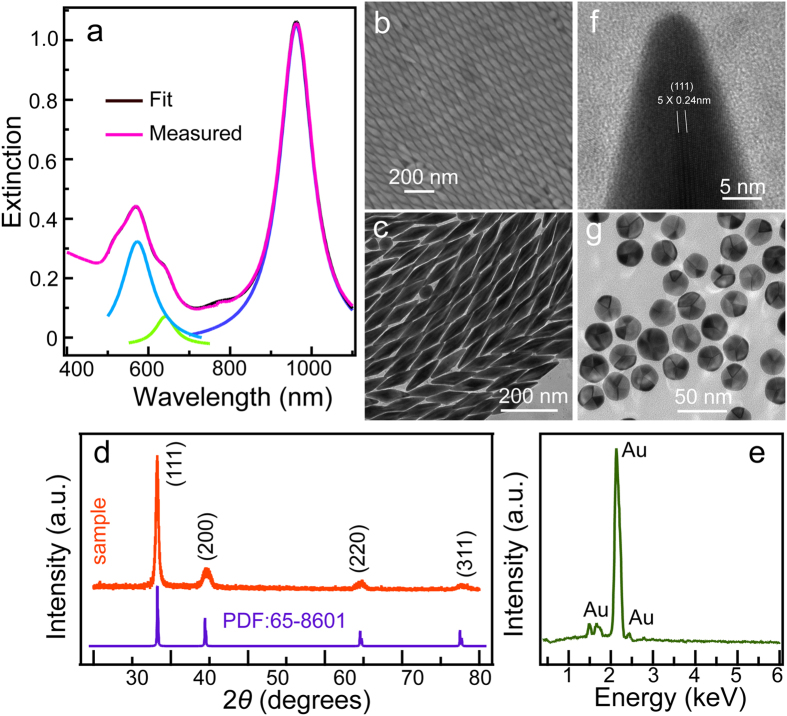
(**a**) Measured extinction spectrum of the typical Au NBP samples. The extinction spectrum was fitted by Lorentzian function with their wavelengths located at 964, 642, and 572 nm, respectively. The fitting coefficient of determination (R^2^) is 0.9998. (**b**–**f**) SEM, TEM, XRD patterns, EDX, and HRTEM of the prepared Au NBP sample, respectively. (**g**) TEM images of the Au nanospheres that were oxidized from Au NBPs.

Au NBPs were prepared through a seed-mediated method, in which H_2_PtCl_6_ were introduced in both seed and growth solutions (for details see experimental section). Figure 1a illustrates the typical extinction spectrum of our Au NBP samples. The Au NBPs distinctly show two plasmon resonance peaks, assigning to a longitudinal plasmon wavelength located at 964 nm and a transverse plasmon wavelength at 572 nm, respectively. The peak intensity ratio between longitudinal and transverse wavelength was determined to be 2.4:1, suggesting a relative high yield determined by the number percentage. In addition, the FWHM value of the longitudinal wavelength was determined to be 96 nm by fitting the experimental extinction spectrum. The relative small FWHM value confirms that the Au NBPs are uniform in size. A relative small amount of byproduct (mainly nanospheres) are also produced, evidenced by the weak plasmonic band fitted to be 642 nm. Scanning and transmission electron microscopy (SEM and TEM) characterizations also verify the formation and high yield of Au NBPs (Fig. 1b,c). Apparently, the as-prepared Au NCs exhibit bipyramid-like morphology formed by two pyramids that share with the same base. The Au NBPs have a uniform size with a length of 173.0 ± 3.7 nm between two apexes and a width of 48.9 ± 3.5 nm measured from TEM images. The aspect ratio is 3.55 ± 0.28 based on the measured sizes. Furthermore, the yield of Au NBPs reaches up to 84.6%. Figure 1d exhibits the X-ray diffraction (XRD) patterns conducted on the as-prepared Au NCs. Four prominent peaks indexed as a cubic structure of Au (Fig. 1d). No diffraction peak contributed to Pt was detected, undoubtedly confirming the formation of pure Au nanostructures. The composition was also obtained by energy dispersive X-ray spectroscopy (EDX) on SEM (Fig. 1e). The NCs are mainly composed of Au (99.05%), with a trace of Ag (0.95%, the peak intensity is too low to display) and Pt (0%). ICP-MS measurements further confirm the pure Au with the atomic percentage for Au, Ag, and Pt of 98.79%, 1.21%, and 0%, respectively. Furthermore, the lattice fringes were measured to be 0.24 nm, assigned to be {111} facets of Au NCs (Fig. 1f). The corresponding electron diffraction pattern is assigned to typical twined crystals, indicating the twined-crystals Au NBP that is similar to the previous reports (Fig. S1a)^7^. Moreover, we also oxidized Au NBPs into Au nanospheres (Fig. S1b). TEM of the Au nanospheres clearly show five boundaries, revealing that the obtained Au NBPs have pentagonally bases (Fig. 1g). These structural feature was previously observed^19^,^20^, but this is the first work that can directly prove in visual.

**Figure 2:**
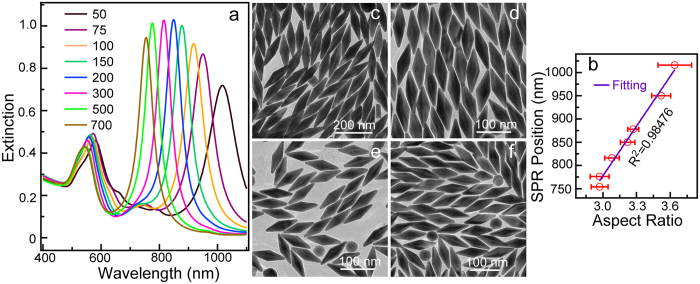
(**a**) Extinction spectra of the Au NBP samples grown by changing the volume of seed solution. (**b**) Variation of the longitudinal plasmon wavelengths as the function of the aspect ratio of Au NBPs. The data points of y-coordinate were extracted from the curves exhibited in (**a**) The x-coordinate data were obtained by measured at least 200 NCs for each sample from TEM images. These data points were linearly fitted. (**c**–**f**) TEM images of the obtained Au NBPs grown from the injection of 50, 150, 300, and 700 μL seed solution.

Synthetically tuneable LSPR properties is the most attractive feature of the anisotropic Au NBPs. The growth parameters during the growth process are critical in their final sizes (aspect ratio), shapes, yields, and therefore their LSPR properties. Usually, the seed-to-Au(III) molar ratio in the growth solution has a significant effect on their sizes^21^. In our work, the sizes of the Au NBPs decrease with the increasing volume of seeds at a given concentration of Au(III). The aspect ratio become low, leading to a blueshift in plasmonic resonance peak. Figure 2a shows the extinction spectra grown by varying the seed-to-Au(III) molar ratio. The molar ratios were tuned by varying the seed volume at the fixed concentration of Au(III) at 0.4 mM (40 mL). The longitudinal plasmon wavelength located at 1018 nm if 50 μL seed solution was injected. As the seed solution increased to 700 μL, the longitudinal plasmonic wavelength gradually blueshifts to 754 nm with FWHM of 59 nm. We plotted the longitudinal plasmon wavelength as the function of the volume of seed solutions to reveal more clearly the peak position variations (Fig. S2). The longitudinal plasmonic peak shows a obvious blueshift as the decrease of the seeds added into growth solutions. It is well known that the amount of added seeds will essentially change the aspect ratios of Au nanostructures which eventually change the longitudinal plasmonic peaks. We therefore also exhibit the variation of longitudinal peaks as the function of their aspect ratios (Fig. 2b). The longitudinal SPR wavelength became linearly redshift with the increase of the aspect ratio of Au NBPs. TEM imaging demonstrates the size and aspect ratio changes (Fig. 2c–f). The lengths vary from 198.7 ± 9.6 nm to 107 ± 4.7 nm if the seed volume increased from 50 to 700 μL. Simultaneously, the width also decrease from 54.9 ± 4.3 nm to 36.1 ± 1.3 nm. Their average aspect ratios thus change from 3.64 to 2.97, causing the blueshift in plasmonic resonance properties.

**Figure 3:**
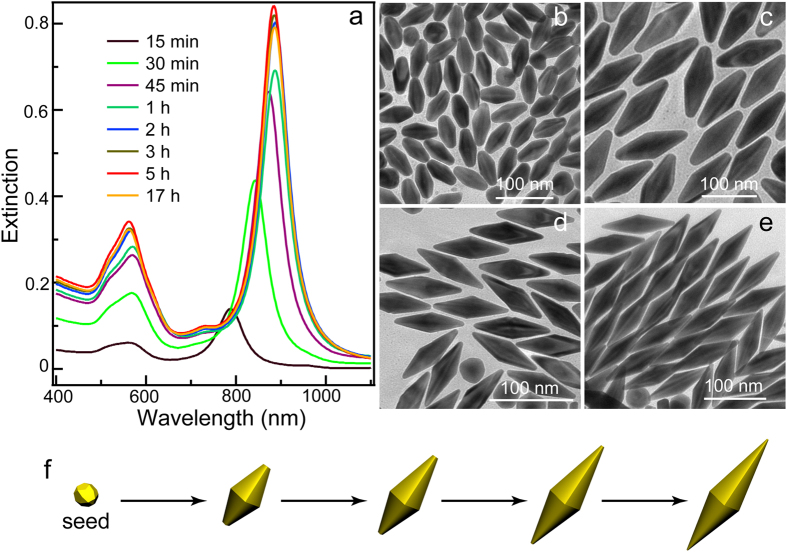
(**a**) Time-dependent extinction spectral variations of the Au NBP samples (100 μL seed solution). (**b**–**e**) TEM images of the obtained Au NBPs collected at 15 min, 30 min, 1 h, and 5 h, respectively. (**f** ) Schematic illustrating the growth process of Au NBPs as the reaction time prolonging.

Intriguingly, we found that the morphology and plasmonic properties of our Au NCs is highly time-dependent. For Au NBP samples grown from 100 μL seed solution, there are steadily redshifts in longitudinal plasmonic peaks as the react time was prolonged (Fig. 3a). Moreover, the intensity also show a gradual increase. Both the plasmonic peak and the intensity become steady. It is well-known that the plasmonic properties of Au NCs are sensitive to the size and morphology. To explain the reaction time-dependent plasmonic variations, TEM images were acquired. After the growth were initiated for 15 min, truncated Au NBPs were observed with a size of (54.0 ± 2.7) × (27.2 ± 1.0) nm (Fig. 3b). For the products obtained at 30 min, the longitudinal size increase to 93.8 ± 4.7 nm (Fig. 3c). The tips of the truncated Au NBPs simultaneously become much sharper when the growth was proceeded for 1 h (Fig. 3d). The sizes of those Au NBPs also increase to (125.2 ± 5.7) × (39.0 ± 2.3) nm. Fig. 3e displays that almost all of the products are Au NBPs with sharp tips. The length and the width of the ideal NBPs further increase to (130.3 ± 3.2) and (39.4 ± 2.0) nm, respectively. The change of shape and size is illustrated in Fig. 3f. The Au NCs grown into truncated Au NBPs at the first stage, which gradually become sharper and sharper. The length and width also simultaneously become larger and larger. Ideal Au NBPs are successfully obtained after about 5 h.

**Figure 4:**
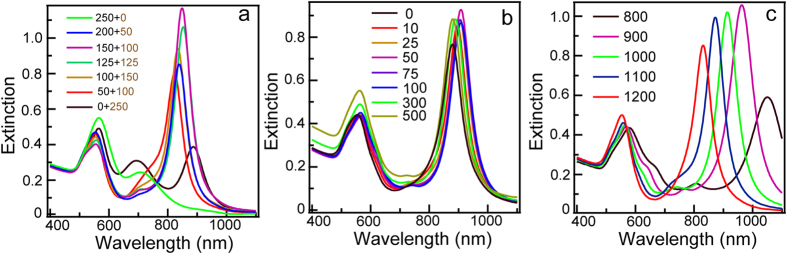
Extinction spectral variations of Au NBP samples growth under different condition. (**a**) The amount of H_2_PtCl_6_ during seed preparations. The number of annotations refers to the volume of HAuCl_4_ (black numbers) and H_2_PtCl_6_ (brown numbers). (**b**) The amount of H_2_PtCl_6_ in growth solutions. (**c**) The amount of NaBH_4_ during seed preparations. The concentration of both H_2_PtCl_6_ and NaBH_4_ were fixed at 0.01 M. The units of annotations in (**a**–**c**) are μL.

To find the optimal growth condition and study the growth mechanism for the synthesis of Au NBPs, we conducted systematical investigations on the variations of extinction spectra and morphologies under different growth conditions. Previously, Ag^+^ have been extensively employed to grow anisotropic Au nanorods^22^. Intriguingly, introduction of H_2_PtCl_6_ during growth process results in Au NBPs, a completely different morphology. To further recognize the role of H_2_PtCl_6_, Au NCs were prepared under the same growth condition except that H_2_PtCl_6_ is absent. SEM images reveal that the sample show rod-like shape (Fig. S3). Almost no Au NBPs were detected during SEM imaging process. These results clearly verify that H_2_PtCl_6_ plays a critical role for the formation of Au NBPs. It is well known that seed-mediated method can divide into two separate steps, involving seed preparation and growth process, which can control the final morphology. In this regard, we first gave a detailed study on the effect of H_2_PtCl_6_ during seed preparation. The molar ratios were increased by increasing the amount of H_2_PtCl_6_ during the seed preparation while keeping the total metal precursors (H_2_PtCl_6_ and HAuCl_4_) at a constant of 2.5 × 10^−6^ mole (Fig. 4a). The extinction spectrum exhibits a weak plasmonic band at 706 nm and a much stronger peak at 566 nm when the seeds with a size of ~4 nm were prepared by only reduced HAuCl_4_ (Fig. S4a). The intensity ratio between the longitudinal and transverse mode even become as low as 1:2.4, which obviously indicates the quite low yield of anisotropic Au NCs. TEM images show that these nanostructures contain Au nanoparticles with small amount of Au nanorods (Fig. S5a). To our surprise, the plasmonic behaviors change dramatically when H_2_PtCl_6_ is introduced during the synthesis of seeds. The intensity ratios between longitudinal and transverse modes are gradually increased to the highest up to 3.0:1 when the ratio of HAuCl_4_ to H_2_PtCl_6_ reach to 1.5:1. The seeds formed at this condition were also characterized by TEM imaging (Fig. S4b). They have a much well monodisperse compared to Au seeds obtained by just reduced HAuCl_4_. However, the intensity ratio between longitudinal and transverse modes suffer a decrease if H_2_PtCl_6_ was further increased. When the seeds was prepared by reduced only H_2_PtCl_6_ in the absence of HAuCl_4_, the extinction spectrum of the obtained Au NCs shows three broad plasmonic bands at 565, 693, and 887 nm resulting from the mixture of Au nanospheres, Au nanorods, and Au NBPs, in which Au NBPs present in a low yield (Fig. S5b). The introduction of H_2_PtCl_6_ in growth solution also have influence on the growth of Au NBPs. Extinction spectrum reveals that much higher yield was achieved even just small amount of H_2_PtCl_6_ (5.0 × 10^−7^mole) was added. Experimentally, there is no obvious improvement if we further increase the concentration of H_2_PtCl_6_ (Fig. 4b). We therefore fixed the ratio of H_2_PtCl_6_:HAuCl_4_ at 1:35 in growth solution. Moreover, it has been accepted that the seed preparation is the most sensitive and important in seed-mediated method. The effect of the amount of NaBH_4_ was therefore investigated. The peak intensity ratio between longitudinal and transverse modes is 2.4:1, the most highest value, when 900 μL NaBH_4_ (0.01M) was added (Fig. 4c). We also investigated the effects of Ag^+^ and CTAB, which play a vital role in the synthesis of anisotropic Au NCs. At low concentration of Ag(I) (<0.023 mM), the peak intensity is relative low (black line in Fig. S6a), suggesting quite low yield of anisotropic Au NCs while the concentration of impurity is high (Fig. S6b). The intensity ratio between longitudinal and transverse mode reaches the highest to 2.0:1 when Ag^+^ (4.0 × 10^−6^ mole) was introduced. Such results clearly reveal that the existence of Ag^+^ with proper amount facilitate the growth of Au NBPs (Fig. S6a). In addition, CTAB is more suitable for the growth of high yield of Au NBPs in comparison to cetyltrimethyammonium chloride (CTAC) due to the different affinities of CTAB and CTAC, that is, Br^−^>Cl^−^ (Fig. S7)^10^.

From the above results together, we can get the following points: (1) In the presence of Ag^+^, Au nanorods covered by {110} and {111} facets can be grown when pure HAuCl_4_ was reduced to form Au seeds. (2) The NCs grown into Au NBPs covered by {111} facets if the seeds was formed by co-reduction of HAuCl_4_ and H_2_PtCl_6_ even if there is no H_2_PtCl_6_ in growth solution. Slightly tuning the amount of H_2_PtCl_6_ in growth solution can just improve the yield of Au NBPs to some content. (3) Ag^+^ is critical for the formation of the anisotropic Au NCs including Au nanorods and Au NBPs. Therefore, we proposed a possible growth mechanism for Au NBPs. As we all know, two stages are involved including seed preparation and growth processes during seed-mediated method, which will determine the final shape. Theoretically, the ultrafine seed nanoparticles are covered by low index facets {111}, {110}, and {100}. These facets have a inhomogeneous surface energy and the order is {110} > {100} > {111}^23^. We can obtain nanostructures with different shape causing by the different growth rate along different facets covered on the seed nanoparticles, which we can tune by changing many growth parameters. (1) The presence of silver ions in growth solution is critical for producing anisotropic Au NCs for both Au nanorods and Au NBPs. The Ag^+^ assisted growth mechanism have two main hypotheses involving underpotential deposition^10^,^24^, and selective adsorption of silver bromide on the Au surface^16^,^25^. In both cases, the growth rate on Au{110} facets is suppressed. the newly formed Au atoms in growth solution will therefore packed onto the {100} facets, producing anisotropic Au NCs. (2) Besides metal silver, other metal, such as Pd, has also been reported to deposit on the surface of Au NCs^18^. Here, it is therefore very credible that metallic Pt, reduced from H_2_PtCl_6_, will deposit on the surface of the Au seeds in our methodology. It is possible that the Pt atoms reduced from NaBH_4_ will predominantly deposit on Au{111} facets in the presence of CTAB surfactant, leading to the low growth rate of Au{111} facets in the following growth process. Taken together, both of the foreign H_2_PtCl_6_ in seed and growth solutions and Ag^+^ in growth solution promote the growth of Au NBPs. The Pt deposition, especially on the seed surface, reduces the growth rate of Au{111} facets on seeds. The Ag^+^ in growth solution will subsequently effect the growth rate of Au{110} facets during growth process. These two growth modes together result in the preferentially growth along < 110 > direction and the stop-growth along < 100 > direction, which is different from that of Au nanorods grown from the seeds without Pt atoms. Such difference leads to the formation of Au NBPs.

**Figure 5:**
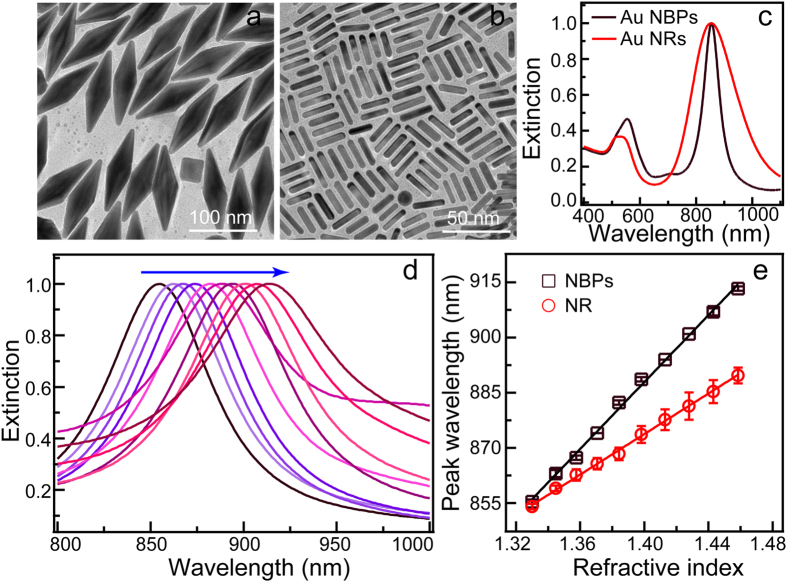
(**a**,**b**) TEM images of the Au NBPs and Au nanorods used in the refractive index sensitivities measurements. (**c**) Normalized extinction spectra of the two aqueous Au NC samples. (**d**) Extinction variations of Au NBPs in water-glycerol mixtures at the range of 800 to 1000 nm. The arrowed line indicates the increase of refractive index. (**e**) Dependence of the longitudinal plasmon wavelength on the refractive index of water-glycerol mixtures. The lines are linearly fitted. The coefficients of the determination (R^2^) for the fitting are 0.9969 and 0.9985 for Au NBP and Au nanorod sample, respectively.

On the basis of the response of LSPR to the change of the surrounding medium, causing the peak shift, plasmonic metal NCs have been utilized in plasmonic spectroscopy^26^. Generally, the index sensitivity for metal NCs increases with longer plasmon bands, larger polarizabilities, and higher curvatures^9^. Au NBP possesses two sharp tips and one basis, providing more sensitive sites with higher curvatures, will be a superior candidate for sensing the index change. In our work, we dispersed the as-prepared ensemble Au NBPs with plasmonic wavelength at 855 nm into liquid medium of different refractive index (Fig. 5a). We also tested the refractive index sensing properties of 854 nm-Au nanorod sample as a comparison (Fig. 5b). Their extinction spectra are shown in Fig. 5c. The refractive indices can be tuned by mixed glycerol (refractive index: 1.4746) into water (refractive index: 1.3334) with various volume percentages. The volume percentages of glycerol in mixtures were varied between 0% to 90% at a step of 10% to ensure the gradually increase of refractive index^27^. The extinction spectral variations reveal that longitudinal plasmonic bands red-shift as the refractive index of the surrounding environment increasing (Fig. S8a–e). Fig. 5d displays the change of longitudinal band of Au NBPs. The variations of the longitudinal modes as the functions of the refractive indices for Au nanorods and NBPs were displayed in Fig. 5e. The RIS of the Au NBP sample was determined to be 464 nm/RIU (refractive index unit). Moreover, the FOM, which is defined as the ratio of the index sensitivity to the FWHM, is 6.44 for Au NBPs. The RIS and FOM of Au nanorod sample is respectively 285 nm/RIU and 1.53. Therefore, the RIS and FOM values of Au NBPs exhibit an increase of 63% and 321% respectively compared to the corresponding values of Au nanorod sample. This resultant sensing properties undoubtedly indicate that the Au NBPs is an superior candidate in sensing applications.

In summary, we first employed H_2_PtCl_6_ as key shape-directing agent to induce the growth of high-yield Au NBPs through a seed-mediated method. H_2_PtCl_6_ with optimized quantity both during the seed preparation and in the growth solution plays critical role for the formation of Au NBPs with sharp edges. In addition, we also investigated other growth factors, such as the amount of NaBH_4_ during seed preparation process, the Ag^+^, and the CTAB surfactant, to obtain the optimal growth condition. More importantly, the size and the longitudinal plasmon band can be controlled by changing the added seed volume, the reaction time, and the volume of NaBH_4_ in seed solution. Moreover, Au NBPs show much larger sensitivity than those of Au nanorods in refractive index. Therefore, we believe that our synthetic methodology provides a new routine to growth Au NBPs and also opens a new way to preparing metal NCs with facile morphology by employing new foreign species.

## Methods

### Synthesis of Au NBPs

The Au NBPs were prepared through a modified seed-mediated method. Typically, the seed solution was prepared through the reduction of HAuCl_4_ (0.01 M, 150 μL) and H_2_PtCl_6_ (0.01 M, 100 μL) with ice-cold NaBH_4_ (0.01 M, 900 μL) in aqueous CTAB (0.1 M, 9.75 mL) solution. The as-prepared seed solution (100 μL) were injected into the growth solution prepared by the sequential addition of HAuCl_4_ (0.01 M, 1.75 mL), H_2_PtCl_6_ (0.01 M, 50 μL), AgNO_3_ (0.01 M, 400 μL), HCl (1 M, 800 μL), and AA (0.1M, 320 μL) into aqueous CTAB (0.1 M, 40 mL). The nanostructures were centrifugated twice for further use.

### Material characterization

SEM images were obtained on an FESEM Hitachi S4800 microscope. TEM imaging was carried out on an FEI Tecnai G^2^ 20 microscope operating at 200 kV. XRD patterns were acquired on Philips X’ Pert system equipped with Cu K α radiation (λ = 1.5419 Å, scanning rate = 1.0°/min). The HRTEM were taken on Tecnai G^2^ 20 S-TWIN operated at 200 kV accelerating voltage. XPS was measured on a Thermo ESCALAB 250 system. The extinction spectra of the Au NCs were acquired on a Hitachi U-3900 with cuvettes with a 0.5-cm optical path length. ICP-AES was conducted by Optima 5300 DV (Perkin Elmer).

## Additional Information

**How to cite this article**: Fang, C. *et al*. Facile Growth of High-Yield Gold Nanobipyramids Induced by Chloroplatinic Acid for High Refractive Index Sensing Properties. *Sci. Rep.* **6**, 36706; doi: 10.1038/srep36706 (2016).

**Publisher’s note:** Springer Nature remains neutral with regard to jurisdictional claims in published maps and institutional affiliations.

## Supplementary Material

Supplementary Information
