# Supplementary material for: Facile Growth of High-Yield Gold Nanobipyramids Induced by Chloroplatinic Acid for High Refractive Index Sensing Properties
Source: Sci Rep. 2016 Nov 14;6:36706. doi: 10.1038/srep36706 (PMC5107934; doi:10.1038/srep36706)
Supplement: Supplementary Information [file srep36706-s1.pdf]

Supporting Information

Facile Growth of High-Yield Gold Nanobipyramids Induced by Chloroplatinic Acid for High Refractive Index Sensing Properties

Caihong Fang*, Guili Zhao, Yanling Xiao, Jun Zhao, ZiJun Zhang, and Baoyou Geng*

College of Chemistry and Materials Science, The Key Laboratory of Functional Molecular Solids, Ministry of Education, Anhui Laboratory of Molecular-Based Materials, Anhui Normal University
Wuhu in Anhui Province 241000 (China)

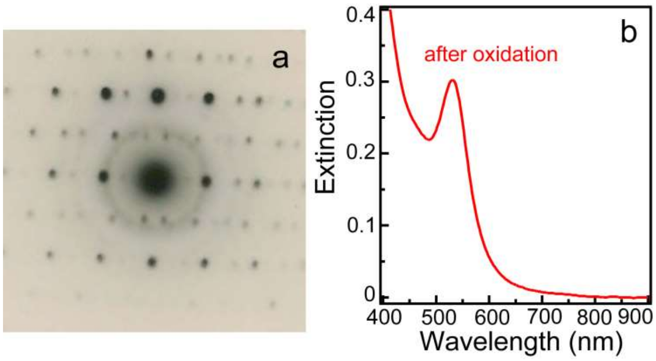

Fig. S1 (a) Selected area electron diffraction pattern taken on the Au NBP corresponding to Figure 1 f in text. (b) Extinction spectrum of the Au NCs oxidized from Au NBPs. The Au NBPs were oxidized with H_2O_2. In a typical experiment, H_2O_2 (30wt%, 200 μL) and HCl (1 M, 100 μL) was dropped into the Au NBPs (40 mL) without any treatment. The extinction spectra were acquired from time to time until the absence of the longitudinal band.

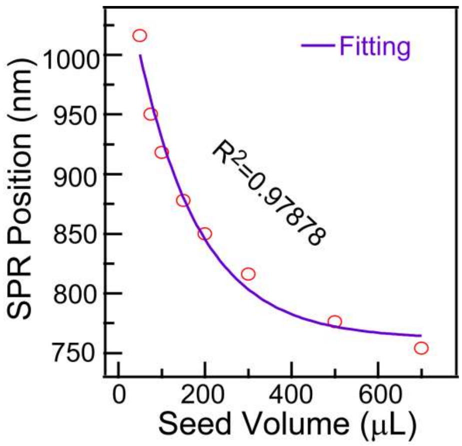

Fig. S2. Variation of the longitudinal plasmon wavelengths as the function of the volume of seed solutions. The data points were extracted from the curves exhibited in Fig. 2a. These data points are fitted by exponential function.

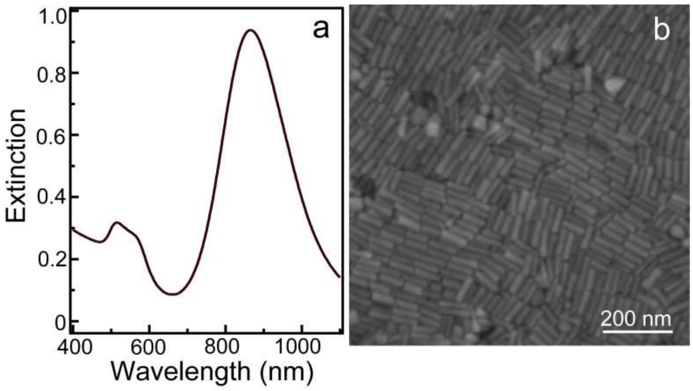

Fig. S3 Extinction spectrum (a) and SEM image (b) of the Au NCs grown from the condition similar to our typical growth parameters but without the addition of H_2PtCl_6 both during the seed preparation and the growth solution.

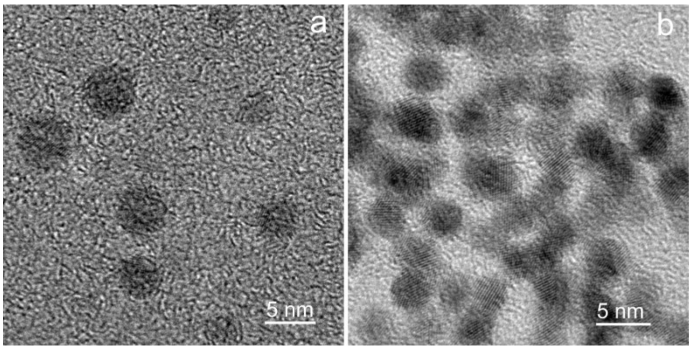

Fig. S4 TEM images of the seeds prepared by reduction of $HAuCl_4$ (a) and the co-reduction of $HAuCl_4$ and H_2PtCl_6.

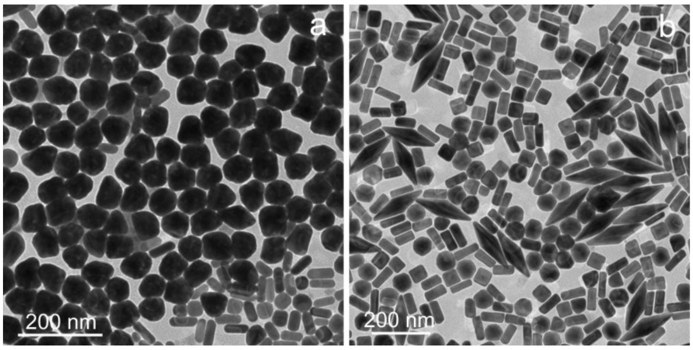

Fig. S5 TEM image of the Au NCs obtained by the seed solution that is prepared by just reduced the $HAuCl_4$ (a) and H_2PtCl_6 (b).

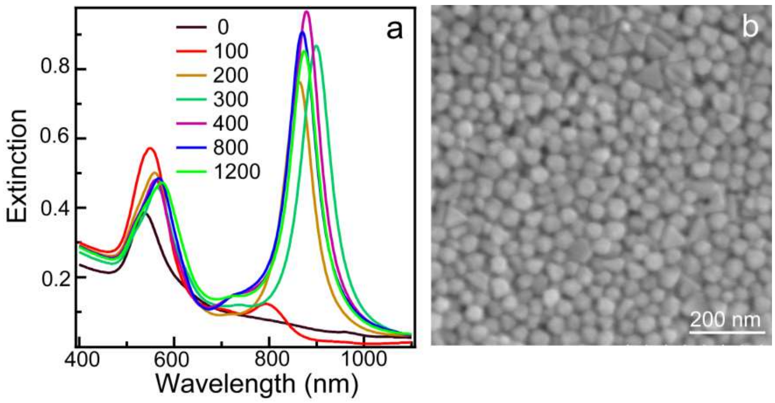

Fig. S6 (a) Extinction spectral variations of Au NBP samples growth under different amount of Ag^+ added in the growth solution. The unit of Ag^+ is μL with concentration of 0.01 M. (b) SEM image of the Au NCs corresponding to the black line in a which is grown in the growth solution without the addition of Ag^+.

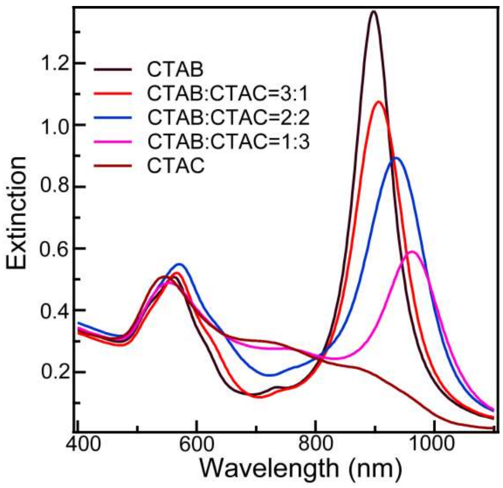

Fig. S7 (a) Extinction variations of Au NCs obtained under the mixture of CTAC and CTAB. The concentration of CTAC and CTAB was fixed at 0.1 M. The ratio of CTAB to CTAC is this figure refers to the volume ratio. The total volume of the surfactant solution is 40 mL. It clearly that the longitudinal plasmonic band become weaker and weaker as the increase of the volume ratio of CTAC. The longitudinal mode even cannot distinguished if CTAB is replaced with CTAC, indicating there is almost no anisotropic Au NCs as the product if CTAC was used as the surfactants.

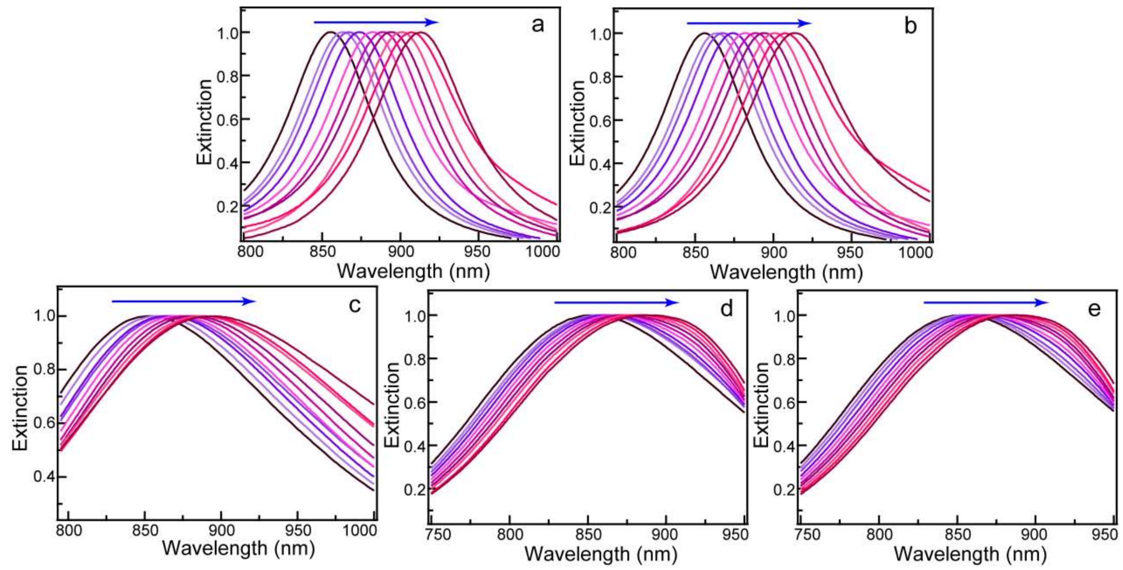

Fig. S8 The extinction spectral changes of Au NBPs (a and b) and nanorods (c−e) in water-glycerol mixtures. The extinction spectra were normalized. The arrowed lines indicate the increase of refractive index.
